# MicroRNA-193a inhibits breast cancer proliferation and metastasis by downregulating WT1

**DOI:** 10.1371/journal.pone.0185565

**Published:** 2017-10-10

**Authors:** FeiYan Xie, Sumayyah Hosany, Shen Zhong, Yang Jiang, Fen Zhang, LiLi Lin, XiaoBo Wang, ShenMeng Gao, XiaoQu Hu

**Affiliations:** 1 Wenzhou Medical University, Wenzhou, Zhejiang, People’s Republic of China; 2 Laboratory of Internal Medicine, The First Affiliated Hospital of Wenzhou Medical University, Wenzhou, Zhejiang, People’s Republic of China; 3 Department of Surgical Oncology, The First Affiliated Hospital of Wenzhou Medical University, Wenzhou, Zhejiang, People’s Republic of China; University of South Alabama Mitchell Cancer Institute, UNITED STATES

## Abstract

In many cancers, microRNA-193a (miR-193a) is a suppressor miRNA, but its underlying anti-oncogenic activity in breast cancer is not known. In this study, we found decreased miR-193a (specifically, miR-193a-5p) expression not only in breast cancer cell lines but also in breast cancer tissues as compared with the adjacent non-tumor tissues. Ectopic miR-193a overexpression inhibited the proliferation, colony formation, migration, and invasion of MDA-MB-231 and BT549 cells. miR-193a reduced Wilms’ tumor 1 (*WT1*) expression and repressed luciferase reporter activity by binding *WT1* coding region sequences; mutation of the predicted miR-193a binding site abolished this effect. miR-193a and *WT1* expression were significantly inversely correlated in breast cancer tissues. Importantly, the anti-cancer activity induced by miR-193a was partially reversed by *WT1* overexpression, indicating an important role for *WT1* in such activity related to miR-193a. Our results reveal that miR-193a-*WT1* interaction plays an important role in breast cancer metastasis, and suggest that restoring miR-193a expression is a therapeutic strategy in breast cancer.

## Introduction

Breast cancer is a clinically heterogeneous disease. Aggressive disease is diagnosed in approximately 10–15% of cases, and distant metastases are developed within 3 years of the initial diagnosis. However, another typical occurrence is distant metastases that manifest at 10 years or more after the initial diagnosis [1]. Therefore, patients face the risk of developing metastasis after initial diagnosis. Defining a cure and assessing metastasis risk factors is difficult, as breast cancer metastasis is of a heterogeneous nature. Therefore, research should focus on molecular mechanisms and therapeutic methods against breast carcinogenesis and metastasis that are more precise so that patient survival and quality of life can be improved.

MicroRNAs (miRNAs) are single-stranded, noncoding RNAs that inhibit gene expression post-transcriptionally by degrading the target mRNA and blocking translation [2]. miRNAs act as major regulators in both human cancer initiation and progression, including that of breast cancer [2, 3]. Oncogenic miRNA upregulation and tumor-suppressive miRNA downregulation can result in malignant proliferation, invasion, and metastasis [4–6]. Pre-miR-193a can generate miR-193a-3p and miR-193a-5p, which are dominant and passenger arms, respectively. miR-193a-3p can suppress tumor development in humans by silencing *SRSF2*, *HIC2*, *HOXC9*, *PSEN1*, *LOXL4*, *ING5*, c-*KIT*, *PLAU*, and *MCL1* [7–18]. In addition, miR-193a-5p can suppress tumor development by modulating cancer cell growth [16, 18]. In addition, miR-193a suppresses the growth, migration, and invasion of breast cancer cells [19]. However, the molecular mechanism of the anti-cancer ability of miR-193a in breast cancer is not known.

The *WT1* gene was originally deemed a suppressor gene in Wilms’ tumor of the kidneys. However, it was subsequently revealed that *WT1* acts as an oncogene in leukemia, lung cancer, breast cancer, and glioblastoma [20]. The *WT1* gene encodes a transcriptional regulator, a zinc finger transcription factor, and RNA-binding protein that can activate or inhibit numerous target genes, leading to proliferation, differentiation, and apoptosis [21]. Therefore, elucidation of the *WT1* regulatory mechanism in breast cancer is important.

Here, we report interaction between miR-193a and *WT1* in breast cancer. miR-193a was decreased in breast carcinoma tissues as compared to the paired adjacent non-cancerous tissues. We found that miR-193a overexpression inhibited the migration and invasion of breast cancer cells by modulating *WT1* expression. Additionally, *WT1* overexpression partially prevented metastasis inhibition induced by miR-193a, suggesting that *WT1* plays an important role in miR-193a anti-metastasis activity. Therefore, the restoration of miR-193a expression might be a novel strategy for improving survival in breast cancer.

## Materials and methods

### Patients and specimens

We obtained breast tumor tissues and the paired normal adjacent tissues from patients without preoperative chemotherapy, hormone therapy, or radiotherapy who had undergone tumor resection at the Department of Surgical Oncology of the First Affiliated Hospital of Wenzhou Medical University between 2012 and 2015. All patients provided written informed consent. We acquired demographic and other clinical variables from the tumor registry database system. The First Affiliated Hospital of Wenzhou Medical University clinical research ethics committee approved the study protocol.

### Cell culture

The MDA-MB-231 and BT549 breast cancer cell lines were purchased from Shanghai Cell Bank (Shanghai, China) and were cultured in RPMI 1640 medium (Gibco, Grand Island, NY, USA) supplemented with 10% fetal bovine serum (FBS, Sigma-Aldrich, St. Louis, MO, USA) and 100 units/mL penicillin and streptomycin in a humidified atmosphere of 5% CO_2_ at 37°C.

### Plasmid construction

To construct the plasmid expressing miR-193a, we amplified the primary sequence of hsa-pre-miR-193a and its flanking regions using specific primer pairs(S1 Table), and then cloned them into pcDNA3.1(-) vector (Invitrogen, Carlsbad, CA, USA) and lentivirus vector pLVX-IRES-ZsGreen1 (Clontech, Palo Alto, CA, USA) to produce LVX-miR-193a. To construct the plasmid expressing *WT1*, we synthesized a human *WT1* isoform [EX5(-)KTS(-), NM_000378] coding sequence (CDS)(S1 Text) directly (GENEWIZ, Suzhou, China) and then cloned it into pcDNA3.1(-) vector (Invitrogen) and retrovirus vector pMSCV-puro (Clontech). The human *WT1* isoform CDS contained a predicted miR-193a-5p target site (AAGACCCA) located in sequence 1538–1545, and not in the 3ʹ untranslated region (3ʹ UTR); the CDS was PCR-amplified and cloned into pMIR-REPORT vector (Ambion, Dallas, TX, USA) and designated pMIR-WT1CDS. We generated a miR-193a-5p binding site mutation in the CDS using a site-directed mutagenesis kit (Agilent Technologies, Palo Alto, CA, USA). S1 Table lists the primer sequences used. We confirmed all constructs via sequencing.

### Luciferase activity

We seeded 1 × 10^5^ HEK293T cells per well in 24-well plates. After 24-h growth, the cells in each well were transiently cotransfected with 100 ng pMIR-REPORT plasmid containing 10 ng internal control vector pRL-SV40 (Promega, Madison, WI, USA), wild-type or mutant pMIR-WT1CDS, and 60 pmol miR-193a mimics or scramble using HiPerFect transfection reagent (Qiagen). After 24 h, we measured firefly and *Renilla* luciferase activity using a Dual-Luciferase Reporter Assay (Promega). The relative luciferase activity was estimated by normalizing firefly luciferase activity to that of *Renilla* for each assay.

### Virus production and cell transfection

Before the 24-h transfection, we plated 4 × 10^6^ HEK293T cells in 10-cm dishes. The cells were cotransfected with pLVX-miR-193a, pLVX-NC (negative control), MSCV-WT1, and MSCV-NC with packaging and envelope vectors. After 48 h, the viruses were harvested from the supernatant, and filtered through 0.45-μm low–protein binding polysulfonic filters (Merck-Millipore, Billerica, MA, USA). We inoculated 2 × 10^5^ MDA-MB-231 or BT549 cells in 6-well plates. Cultures that were approximately 30–60% confluent were transfected with lentivirus pLVX-miR-193a or pLVX-NC, followed by 8 μg/mL polybrene (Sigma-Aldrich) to increase infection efficiency. Subsequently, the cells were transfected with MSCV-WT1 or MSCV-NC. Positive clones were selected via 1-week puromycin (2 μg/mL, MedchemExpress, Princeton, NJ, USA) selection.

### RNA extraction and quantitative reverse transcription–PCR (qRT-PCR)

We isolated total RNA from the cultured cells and patient tissues using TRIzol (Invitrogen) according to the manufacturer’s instructions, and reverse-transcribed it into complementary DNA (cDNA) using an RT kit (TOYOBO, Shanghai). The cDNA template was PCR-amplified using Thunderbird SYBR qPCR mix (TOYOBO) on an ABI PRISM 7500 instrument (Applied Biosystems, Carlsbad, CA, USA). The reactions were incubated in a 96-well optical plate at 95°C for 1 min, followed by 40 cycles of 95°C for 15 sec and 60°C for 1 min. Expression levels were normalized to glyceraldehyde-3-phosphate dehydrogenase (GAPDH), and we calculated relative gene expression levels using the comparative threshold cycle (2^−ΔΔCt^) method. All experiments were performed in triplicate.

### Western blotting

We isolated total proteins from the cultured cells, and detected protein concentrations using a Pierce bicinchoninic acid protein assay kit (BCA Protein Assay Kit). We separated 20 μg protein from each sample using 12% sodium dodecyl sulfate–polyacrylamide gel electrophoresis and transferred them electrophoretically to nitrocellulose membranes (Millipore, Billerica, MA, USA). The membranes were blocked with 5% skimmed milk powder in phosphate-buffered saline (PBS) containing 0.1% Tween 20 at 37°C for 2 h, and then incubated overnight with primary antibodies against *WT1* (1:1000; Cell Signaling Technologies, Beverly, MA, USA) and β-actin (1:5000; Cell Signaling Technologies). Horseradish peroxidase–conjugated anti-rabbit immunoglobulin G (Cell Signaling Technologies) was used as the secondary antibody. Bands were scanned using a densitometer (Bio-Rad, Richmond, CA, USA), and performed quantification using Bio-Rad Image Lab 4.1 software.

### Cell proliferation assay

To assess cell proliferation, we seeded 2 × 10^3^ MDA-MB-231 and BT549 cells per well in 100 μL culture medium in quintuplicate in 96-well plates. We measured the cell proliferation index using the 3-(4, 5-dimethylthiazol-2-yl)-2,5-diphenyl bromide assay (Cell Counting Kit-8, CCK-8, TOYOBO) according to the manufacturer’s instructions at day 1, 2, 3, and 4 after transfection. We measured optical density (OD) at 450 nm using an automated microplate reader (Beckman DU6400 spectrophotometer, Beckman Coulter).

### Cell migration assay

The cell migration assay was performed in vitro using 24-well Transwell chambers. Cells (2 × 10^4^) were seeded in the top chambers, and the bottom chambers were filled with RPMI 1640 medium (600 μL) containing 10% FBS to stimulate migration. After 18-h incubation, the cells were stained with 0.1% crystal violet. The cells that had migrated through the ostioles to the reverse side were counted under a microscope in five pre-determined fields at ×200 magnification. Each assay was performed in triplicate.

### Cell invasion assay

The cell invasion assay was performed using 24-well Transwell chambers. Cells (1 × 10^5^) were seeded in the Matrigel-coated (BD Biosciences, USA) top chambers. The bottom chambers were filled with RPMI 1640 medium (600 μL) containing 10% FBS to stimulate invasion. After 16-h incubation, the cells were stained with 0.1% crystal violet. The cells that had invaded through the Matrigel to the reverse side were counted under a microscope in five pre-determined fields at ×200 magnification. Each assay was performed in triplicate.

### Wound healing assay

Cells (2 × 10^5^) in a 6-well plate were grown to 80% confluence at 37°C in a 5% CO_2_ incubator. The monolayers were scratched with a plastic pipette tip, washed with PBS to remove cell debris, and incubated in serum-free medium for 24 h. We photographed cells within the tagged fields under phase-contrast microscopy (×100 magnification) at 0 h and 24 h after wounding.

### Colony formation assay

We stably transfected MDA-MB-231 or BT549 cells with pLVX-NC or pLVX-miR-193a. After pancreatic enzyme digestion, the cells were counted and we inoculated an average 2000 cells per well in 6-well plates. We changed the medium every 3–4 days. After 2 weeks, the cells were washed using PBS, and fixed for 10 min in cooled methanol. Then, 0.1% crystal violet was added and allowed to incubate for 40 min. Next, the plates were washed with clean water to remove the stain, and the number of colonies formed was counted under ×200 magnification (minimum criterion for a colony = 50 cells).

### Immunofluorescence assay

We seeded 10^5^ cells per mL breast cancer cells onto coverslips and cultured them in a 6-well culture plate for 24 h. The cells were washed in cold PBS and fixed in 2% paraformaldehyde–PBS for 30 min, permeabilized in methanol for 20 min at 4°C, and blocked in 5% bovine serum albumin for 120 min at room temperature. The coverslips were incubated overnight with primary antibody against *WT1* (1:100; Cell Signaling Technologies), followed by incubation with tetramethylrhodamine isothiocyanate–conjugated secondary antibody for 1 h, and then diamidinophenylindole staining. Finally, the coverslips were observed under a fluorescence microscope (×1000 magnification, Olympus BX51 Tokyo, Japan).

## Statistical analysis

The data are reported as the mean ± SD. The Student *t*-test (2-tailed) was used to determine the significance of differences between the groups. The association between *WT1* or miR-193a expression and clinicopathological variables was analyzed using Fisher’s exact test. Two-group comparisons of miR-193a or *WT1* were made using a paired Student’s *t*-test. The statistical analysis was performed using SPSS software (SPSS 22.0, Chicago, IL, USA). P < 0.05 was deemed statistically significant.

## Results

### Study subjects

We enrolled 25 patients according to the inclusion criterion. Table 1 lists the clinical characteristics of the subjects and their miR-193a and *WT1* expression levels.

**Table 1:** miR-193a and WT1 expression levels and the clinical parameters of 25 breast cancer specimens.

Case	Age (years)	Maximum diameter (cm)	Stage	TNM stage	ER	PR	HER2	Ki67	Positive/total lymph node	Histological type	miR-193a expression level	WT1 expression level
1	71	5	III	I	+	+	-	5–8%+	0/16	DCIS	0.147	0.122
2	43	1.8	I	I	40%+	2%+	+++	30%+	0/6	DCIS	0.375	0.698
3	44	5	II	II	+	+	-	3%+	3/26	IDC	7.129	0.117
4	75	3	II	II	-	-	++	80%+	0/12	IDC	3.965	0.481
5	55	1.8	II	II	-	-	+++	40%+	3/10	IDC	2.788	1.520
6	46	1	II	I	-	-	+++	10%+	0/4	DCIS	6.603	0.206
7	51	2.2	II	II	Absent	Absent	Absent	Absent	1/16	Myxoadenocarcinoma	3.992	0.255
8	60	2.2	II	II	95%+	95%+	-	40%+	0/10	IDC	0.000490	0.768
9	36	1.2	II	I	+-	+	-	+-	0/5	IDC	5.146	0.254
10	65	1.5	II	I	+	Partial+	+	5%+	1/12	IDC	0.138	0.007
11	49	3	II	III	80%+	10%+	-	30%+	20/29	IDC	3.1835	3.415
12	43	7	II	II	+	+	-	10–20%+	0/12	ILC	0.045	0.394
13	44	4	I	III	+	+	-	5–10%+	3/16	IDC	0.000794	0.071
14	45	2.4	III	III	+	+	-	10%+	0/20	IDC	11.300	1.204
15	44	2	III	II	10%+	-	+++	40%+	0/10	IDC	5.910	0.184
16	45	5.5	II	III	-	-	+++	30%+	5/15	IDC	0.352	0.055
17	53	2.5	II	II	-	-	+++	20%+	0/6	IDC	0.900	1.344
18	37	1.5	II	I	+	+	-	2%+	0/13	IDC	7.684	0.148
19	56	2	III	II	+	Several+	-	30%+	1/12	IDC	0.089	0.050
20	56	3	III	II	-	-	+++	30%+	0/16	IDC	2.399	0.703
21	41	3.1	II	III	+	+	+++	20%+	4/23	IDC	0.003	2.383
22	52	5	II	III	+	+	-	5–10%+	4/10	ILC	0.014	0.138
23	57	1.7	II	II	+	-	+++	20%+	1/3	IDC	3.226	0.134
24	67	3	I	I	+	+	-	Several+	0/7	DCIS	11.017	0.076
25	57	4	II	II	+	-	++	20–25%+	0/18	IDC	0.005	1.584

TNM, Tumor-node-metastasis

DCIS, Ductal carcinoma in situ

IDC, Invasive ductal carcinoma

ILC, Invasive lobular carcinoma

“-”, negative

“+-”, weak positive

“+”, positive

“++”, moderate positive

“+++”, strong positive

### miR-193a was downregulated in breast cancer tissues

**Fig 1:**
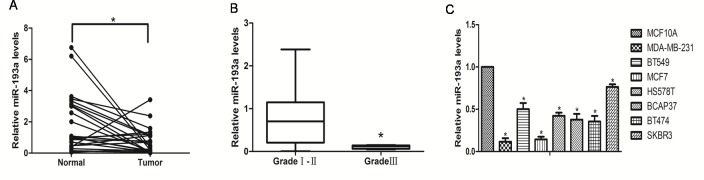
miR-193a expression levels in breast cancer. (A) qRT-PCR analysis of relative miR-193a expression levels in 25 pairs of breast carcinoma tissues (Tumor) and adjacent non-cancerous tissues (Normal). (B) miR-193a levels in advanced breast cancer (stage III) tissues were lower than that in early breast cancer (stage I–II) tissues. (C) qRT-PCR analysis of relative miR-193a expression levels in the MCF-10A cell line and seven breast cancer cell lines. *p < 0.05.

To analyze miR-193a expression in breast cancer, we first detected miR-193a levels in 25 pairs of human breast carcinoma tissues and their adjacent non-cancerous tissues. miR-193a expression was significantly lower in the breast carcinoma tissues than in the paired adjacent non-cancerous tissues (Fig 1A, p = 0.0059). miR-193a expression was much lower in advanced disease (stage III, n = 5) compared to early-stage disease (stage I–II, n = 20) (Fig 1B, p = 0.0277). Next, we measured miR-193a levels in human breast cancer cell lines, and found significantly lower levels of miR-193a expression in the breast cancer cell lines than in the normal breast epithelial cell line MCF-10A (Fig 1C). miR-193a expression was lowest in MDA-MB-231 cells compared with the other breast cancer cell lines. BT549 cells are triple-negative breast cancer cells with high invasive ability. Based on these findings, we selected the MDA-MB-231 and BT549 cell lines for the subsequent experiments.

### miR-193a inhibited breast cancer cell migration, invasion, and proliferation

**Fig 2:**
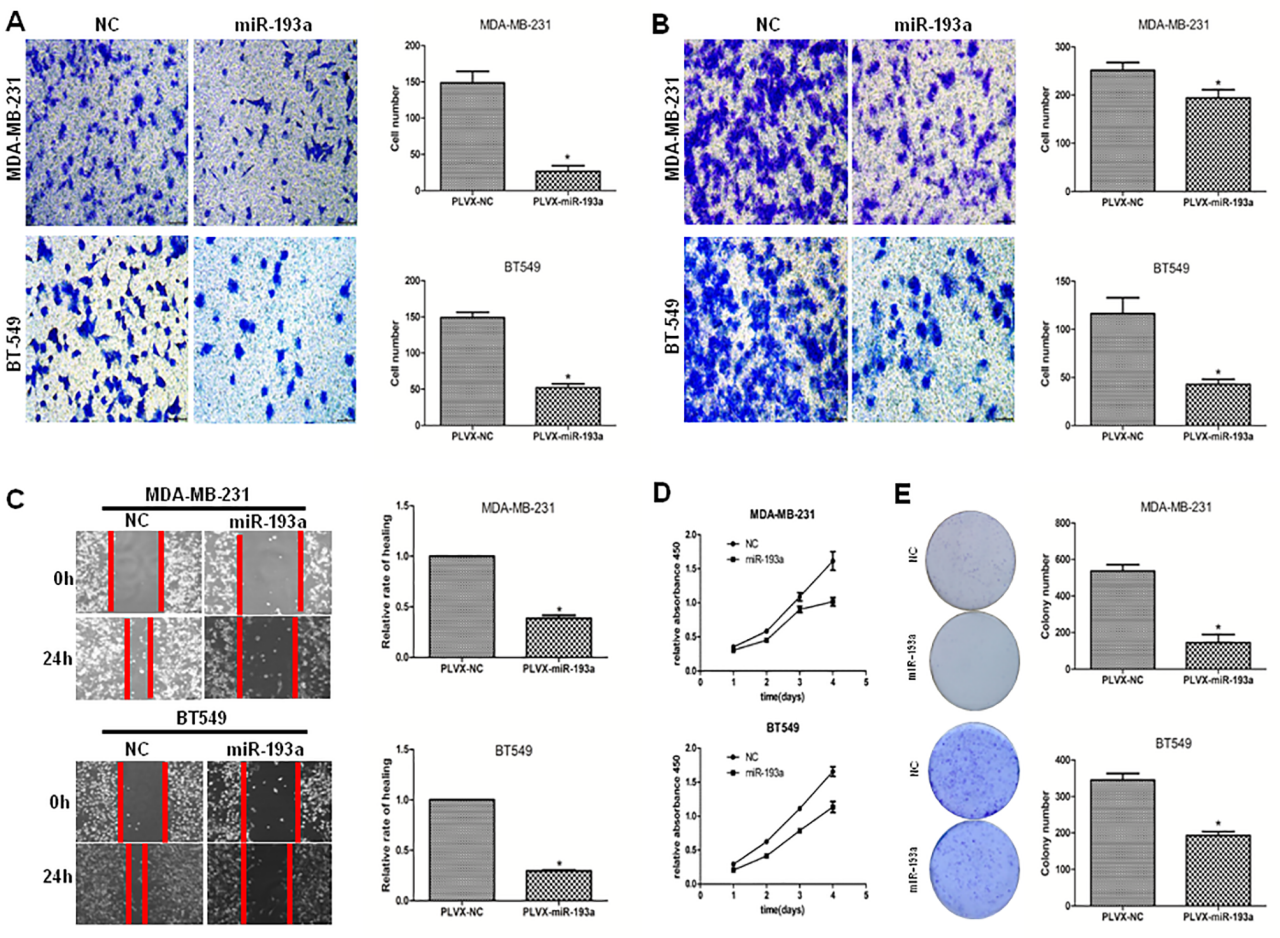
The role of miR-193a in regulating breast cancer cell migration, invasion, and proliferation. (A) Migration assay. (B) Invasion assay. (C) Wound healing assay. (D) Proliferation assay. (E) Colony formation assay. The mean was derived from the cell counts of five fields; each experiment was repeated three times. Representative images of migrated, invaded, or proliferative cells are shown. *p < 0.05.

To determine the biological role of miR-193a in breast cancer, we transfected MDA-MB-231 and BT549 cells with pLVX-miR-193a or the negative control pLVX-NC. miR-193a levels in the MDA-MB-231 and BT549 cells were increased by about 131-fold and 62-fold, respectively, compared to the negative control. miR-193a overexpression inhibited breast cancer cell migration (Fig 2A) and invasion (Fig 2B); restoring miR-193a expression significantly inhibited MDA-MB-231 and BT549 cell motility, proliferation, and colony formation (Fig 2C–2E).

### miR-193a targeted WT1 directly

**Fig 3:**
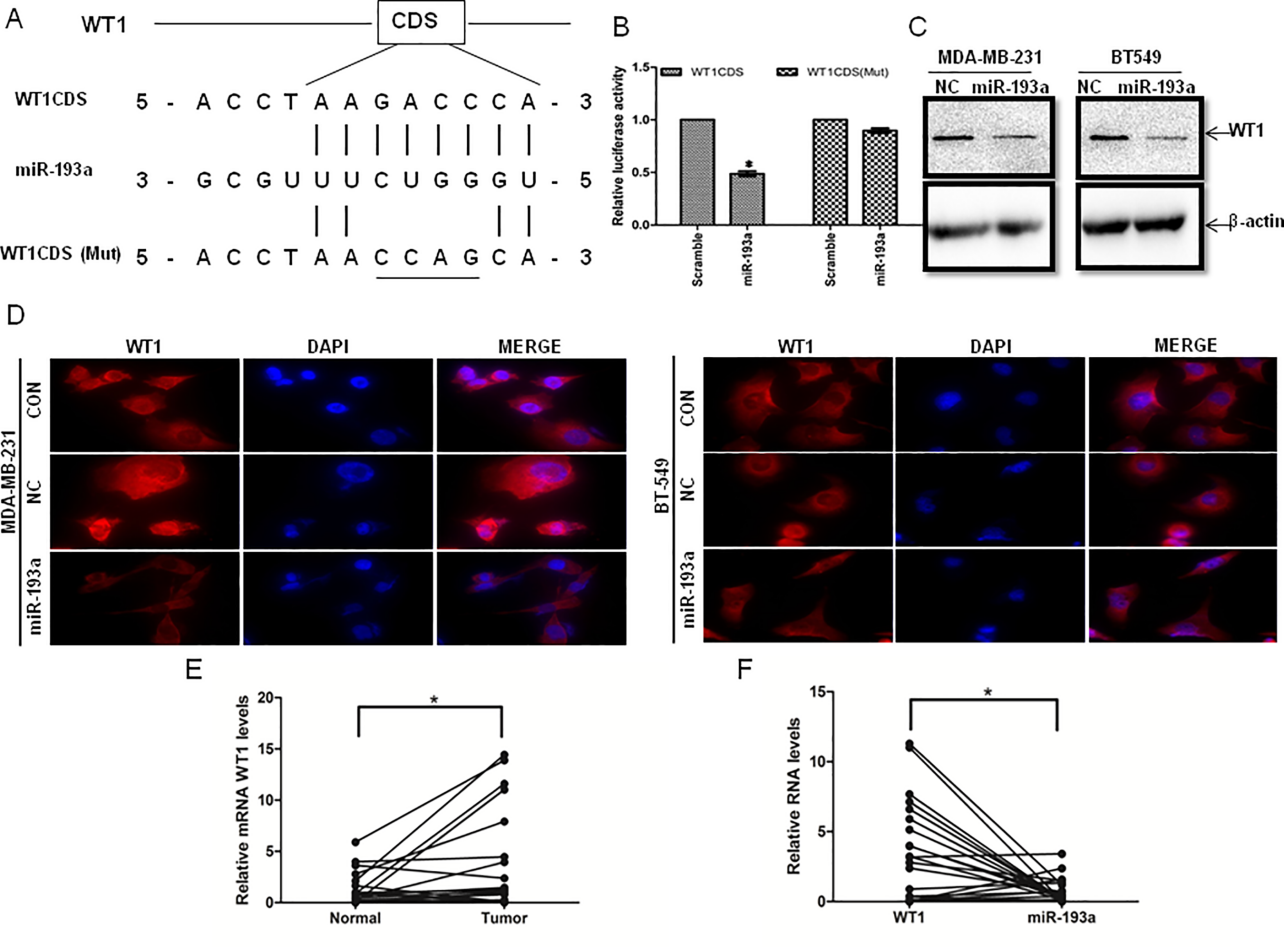
Verification of miR-193a targeting of WT1. (A) Base complementarity between miR-193a and *WT1* mRNA. (B) Luciferase reporter assay of HEK293T cells cotransfected with wild-type (*WT1* CDS) or mutant (*WT1* CDS Mut) pMIR-WT1 CDS and miR-193a mimics or negative control. (C) Western blot analysis of WT1 protein expression in empty vector-negative control (pLVX-NC) cells, or cells overexpressing miR-193a (pLVX-miR-193a). Results are representative of three independent experiments. (D) Immunofluorescence staining for WT1 protein in cells with empty vector-negative control (pLVX-NC) or pLVX-miR-193a. Scale bar = 100 μm. (E) qRT-PCR analysis of relative expression levels of *WT1* in 25 pairs of breast carcinoma tissues (Tumor) and adjacent non-cancerous tissues (Normal). (F) *WT1* was negatively correlated with miR-193a at mRNA level (n = 25). *p < 0.05.

We used miRanda (http://www.microrna.org/microrna) to predict possible target genes to select the possible miR-193a target gene. Among the candidates, *WT1* was selected for further study because it acts as an oncogene in human cancers. The *WT1* CDS containing putative wild-type or mutant miR-193a binding sites was subcloned into pMIR vector to produce wild-type vector (pMIR-WT1CDS) and mutation vector (pMIR-WT1CDS, Mut), respectively (Fig 3A). HEK293T cells were cotransfected with the vectors with miR-193a mimics or negative control. miR-193a overexpression decreased luciferase activity by approximately 50%. However, the mutated putative miR-193a binding site almost abolished the decrease of luciferase activity (Fig 3B).

Western blotting (Fig 3C) and immunofluorescence staining (Fig 3D) showed that in cells transfected with pLVX-193a, WT1 protein was downregulated as compared to transfected with the negative control.

qRT-PCR was conducted on 25 pairs of human breast cancer tissues and their adjacent non-cancerous tissues to validate the correlation between miR-193a and endogenous *WT1* expression in breast cancer. *WT1* expression was significantly upregulated in breast carcinoma tissues compared to that in the adjacent non-cancerous tissues (Fig 3E). When the relative expression of miR-193a was plotted against the relative expression of *WT1* (Fig 3F), it revealed that *WT1* and miR-193a were significantly inversely correlated (R^2^ = 0.1859; p = 0.0314).

### miR-193a downregulated WT1 protein; ectopic WT1 overexpression partially prevented inhibition of metastasis and proliferation induced by miR-193a

**Fig 4:**
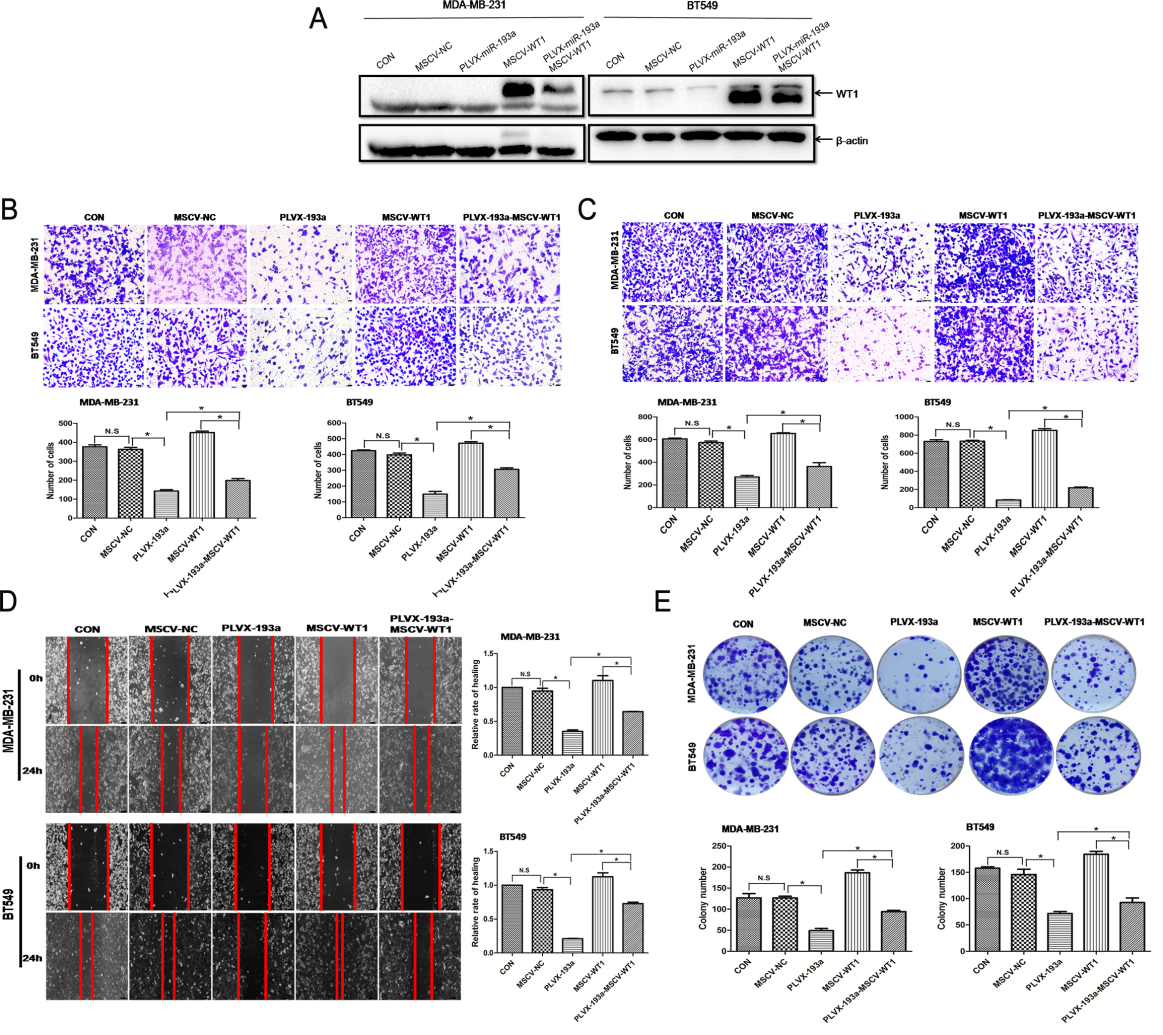
Western blot analysis. (A) WT1 protein expression in MDA-MB-231 and BT549 cells transfected with MSCV-NC, pLVX-miR-193a, MSCV-WT1, or MSCV-WT1 and pLVX-miR-193a. (B) Migration assay, (C) invasion assay, (D) wound healing assay, and (E) colony formation assay of miR-193a–WT1 interaction in regulating breast cancer cell migration, invasion, and proliferation. The mean was derived from the cell counts of five fields; each experiment was repeated three times. Representative images of proliferative cells are shown. *p < 0.05.

To determine whether *WT1* overexpression would prevent metastasis induced by miR-193a, we cotransfected MDA-MB-231 and BT549 cells with pLVX-miR-193a and MSCV-WT1. Cells transfected with MSCV-WT1 had significantly increased *WT1* expression as compared with the negative control (Fig 4A, lane 4 versus lane 2). Furthermore, miR-193a expression slightly weakened *WT1* overexpression (Fig 4A, lane 5 versus lane 4).

As metastasis was inhibited by miR-193a targeting of WT1 protein, we examined whether *WT1* counteracts the anti-metastasis effect induced by miR-193a. We cotransfected MDA-MB-231 and BT549 cells with pLVX-miR-193a and MSCV-WT1 to examine cell migration and invasion. The miR-193a–induced anti-migration (Fig 4B) and anti-invasive (Fig 4C) effects were reduced by WT1 overexpression. *WT1* overexpression also partially prevented the inhibition of motility induced by miR-193a (Fig 4D), and similarly partially blocked the decreased colony formation induced by miR-193a (Fig 4E).

## Discussion

*WT1* is a complex gene with 10 exons, and expresses at least 36 subtypes, each with four zinc fingers [20]. Different roles have been ascribed to *WT1* in carcinogenesis. Although *WT1* was initially identified as a suppressor gene in Wilms’ tumor, it acts as an oncogene in malignancies such as leukemia, glioblastoma, and lung cancer [20]. Its role in breast carcinogenesis is poorly understood. In the early stage of breast cancer development, DNA hypermethylation occurs in the *WT1* promoter and leads to the low *WT1* expression in breast cancer [22]. In breast cancer, higher *WT1* expression is associated with higher histological stage and worse prognosis, and *WT1* acts as an oncogene in breast cancer progression [23]. Our study also shows that the adjacent non-cancerous tissues had low levels of *WT1* as compared with the breast cancer tissue. Although the relationship between *WT1* and breast cancer has been studied, the biological significance of *WT1* signaling in breast cancer has not been completely elucidated.

miRNAs are often abnormally expressed in cancerous tissue and can have cancer gene or tumor suppressor gene characteristics [4, 24]. miR-193a deficiency is present in many cancers [16, 25–29]. We, too, report reduced miR-193a-5p expression in breast cancer tissues compared with the adjacent non-cancerous tissues. miR-193a-3p is a novel tumor suppressor that can inhibit cell cycle progression and proliferation in breast cancer by targeting cell cycle network proteins driven by epidermal growth factor receptor (EGFR) [30]. Our experiments suggest that it is likely that miR-193a-5p is involved in carcinogenesis and acts as a tumor suppressor in breast cancer. One miRNA can target multiple genes, while numerous miRNAs can target the same gene. Consequently, miR-193a may target multiple genes other than *WT1*, which may also have important roles in carcinogenesis. For example, in acute myeloid leukemia, miR-193a can repress c-*KIT* proto-oncogene expression and function as a tumor suppressor silenced by methylation [31]. miR-193a can also act as a tumor suppressor by targeting *ERBB4* (human EGFR 2, HER2) and play a role in inhibiting proliferation and invasion and accelerating the apoptosis of lung cancer cells [14]. Uhlman and colleagues used reverse phase protein array to explore EGFR pathway regulation that involved miRNA, systematically identifying miRNAs that regulated the expression of 26 EGFR pathway proteins. They identified miR-124, miR-147, and miR-193a-3p as tumor suppressors that can inhibit breast cancer proliferation by regulating the cell cycle network protein driven by EGFR [30]. Leivonen and colleagues characterized miRNAs that targeted *HER2* directly, and identified seven novel miRNAs (miR-552, miR-541, miR-193a-5p, miR-453, miR-134, miR-498, miR-331-3p) that regulated the 3ʹ UTR of *HER2* directly [32]. At this point, the most important question therefore is how important this new pathway is in breast carcinogenesis. Here, we found that miR-193a overexpression inhibited the proliferation, migration, and invasion of breast cancer cells. The enforced *WT1* expression successfully, albeit partially, reversed the anti-proliferative, anti-migration, and anti-invasion effects of miR-193a on breast cancer cells, although there are many targets of miR-193a. Our results suggest that the targeting of *WT1* is an important mechanism by which the tumor-suppressive function of miR-193a is exerted. In this study, we have partly elucidated miR-193a regulation of *WT1* in breast cancer, where the mechanism of miR-193a downregulation during carcinogenesis can accelerate cell growth and promote cancer cell diffusion.

Although most studies indicate that miR-193a acts as a tumor suppressor, several other studies suggest that miR-193a is an oncogene and facilitates cancer cell proliferation. For example, knockdown of miR-193a-3p significantly inhibited cell proliferation and colony formation and induced cells into G1 phase arrest by directly targeting PTEN(phosphatase and tensin homolog deleted on chromosome ten), indicating that miR-193a-3p functions as a tumor-promoting microRNA in renal cell carcinoma[33]. Also, Fisher et al. reported that two antitumor agents (retinoic acid and lapatinib) had antimetastatic properties mediated by decreasing the expression of miR-193a, suggesting that miR-193a might be an oncomiR. Overexpression of miR-193a increased proliferation and survival of SKBR3 cells, thereby functioning as a tumor-promoting microRNA. SKBR3(estrogen receptor-negative and HER2+) cells are supposed to be greatly sensitive to the anti-neoplastic action of retinoic acid (ATRA) targeting RARα and lapatinib targeting HER2[34]. Single miRNAs present biological activity by targeting different proteins, which may be differently expressed in cancer cells, resulting in the contradictory activity observed in different cell types. These discrepancies suggest the important role of cellular context in the function of miRNAs. Therefore, more studies are required to shed light on the activity of miR-193a in breast cancer cells.

Many studies have shown that the occurrence and development of breast cancer, including tumor metastasis, are complicated processes in which epithelial–mesenchymal transition (EMT) and mesenchymal–epithelial transition (MET) take place. A regulatory factor, *WT1* can regulate and control EMT or MET in the epithelial cells of different organs. For example, in heart development, *WT1* can activate the *WNT4* gene specifically, subsequently controlling the interstitial cell epithelium. However, in kidney tissues, *WT1* can specifically activate another gene and play a promoter role in MET. In the present study, breast carcinoma tissue samples with higher pathological stages had lower levels of miR-193a expression. These findings imply that miR-193a levels are associated with the degree of tumor cell differentiation. Therefore, we speculate that, miR-193a can affect the specific regulatory factor *WT1* in mammary gland cells, influencing cell transformation and differentiation. In future studies, we will elucidate the downstream of *WT1*.

## Conclusions

Identifying and characterizing miR-193a in breast cancer provides a better understanding of targeted therapy. miR-193a acts as a tumor suppressor by targeting *WT1*, thereby suppressing breast cancer growth and metastasis. The negative correlation between miR-193a expression and *WT1* indicates that patients with breast cancer who have lower miR-193a expression may have higher *WT1* expression, which might contribute to the activation of carcinogenesis and invasion. These results favor a strong correlation between *WT1* and miR-193a, indicating that restoration of miR-193a expression may be a promising strategy for breast cancer clinical therapies and that *WT1* could be a novel biomarker of breast cancer prognosis and diagnosis, and a potential molecular therapeutic target.

## Supporting information

S1 Table qRT-PCR primer sequences. (DOC)

S1 Text CDS of the human *WT1* isoform [EX(-)KTS(-) NM_000378]. The base sequences in red are the target sites complemented by miR-193a-5p.
(DOC)

## References

[1] Weigelt B, Peterse JL, van 't Veer LJ. Breast cancer metastasis: markers and models. Nature reviews Cancer. 2005;5(8):591–602. doi: 10.1038/nrc1670 16056258 10.1038/nrc1670

[2] Bartel DP. MicroRNAs: target recognition and regulatory functions. Cell. 2009;136(2):215–33. doi: 10.1016/j.cell.2009.01.002 19167326 10.1016/j.cell.2009.01.002 PMC3794896

[3] Wang W, Luo YP. MicroRNAs in breast cancer: oncogene and tumor suppressors with clinical potential. Journal of Zhejiang University Science B. 2015;16(1):18–31. doi: 10.1631/jzus.B1400184 25559952 10.1631/jzus.B1400184 PMC4288941

[4] Calin GA, Croce CM. MicroRNA signatures in human cancers. Nature reviews Cancer. 2006;6(11):857–66. doi: 10.1038/nrc1997 17060945 10.1038/nrc1997

[5] Ma L, Weinberg RA. Micromanagers of malignancy: role of microRNAs in regulating metastasis. Trends in genetics: TIG. 2008;24(9):448–56. doi: 10.1016/j.tig.2008.06.004 18674843 10.1016/j.tig.2008.06.004

[6] Nicoloso MS, Spizzo R, Shimizu M, Rossi S, Calin GA. MicroRNAs—the micro steering wheel of tumour metastases. Nature reviews Cancer. 2009;9(4):293–302. doi: 10.1038/nrc2619 19262572 10.1038/nrc2619

[7] Wang J, Yang B, Han L, Li X, Tao H, Zhang S, et al Demethylation of miR-9-3 and miR-193a genes suppresses proliferation and promotes apoptosis in non-small cell lung cancer cell lines. Cellular physiology and biochemistry: international journal of experimental cellular physiology, biochemistry, and pharmacology. 2013;32(6):1707–19. 10.1159/000356605 24356455

[8] Iliopoulos D, Rotem A, Struhl K. Inhibition of miR-193a expression by Max and RXRalpha activates K-Ras and PLAU to mediate distinct aspects of cellular transformation. Cancer research. 2011;71(15):5144–53. doi: 10.1158/0008-5472.CAN-11-0425 21670079 10.1158/0008-5472.CAN-11-0425 PMC3148313

[9] Salvi. A, Conde I, Abeni. E, Arici. B, Grossi. I, Specchia. C, et al Effects of miR-193a and sorafenib on hepatocellular carcinoma cells. Molecular cancer. 2013;12(162). 10.1186/1476-4598-12-162 PMC4029516 24330766

[10] Deng. H, Lv. L, Li Y, Zhang. C, Meng. F, Pu. Y, et al MiR-193a-3p regulates the multi-drug resistance of bladder cancer by targeting the LOXL4 gene and the Oxidative Stress pathway. Molecular cancer. 2014;13(234). 10.1186/1476-4598-13-234 PMC4200202 25311867

[11] Lv L, Deng H, Li Y, Zhang C, Liu X, Liu Q, et al The DNA methylation-regulated miR-193a-3p dictates the multi-chemoresistance of bladder cancer via repression of SRSF2/PLAU/HIC2 expression. Cell death & disease. 2014;5:e1402. 25188512 10.1038/cddis.2014.367 PMC4540198

[12] Deng H, Lv L, Li Y, Zhang C, Meng F, Pu Y, et al The miR-193a-3p regulated PSEN1 gene suppresses the multi-chemoresistance of bladder cancer. Biochimica et biophysica acta. 2015;1852(3):520–8. doi: 10.1016/j.bbadis.2014.12.014 25542424 10.1016/j.bbadis.2014.12.014

[13] Li. Y, Deng. H, Lv. L, Zhang. C, Qian. L, Xiao. J, et al The miR-193a-3p-regulated ING5 gene activates the DNA damage response pathway and inhibits multi-chemoresistance in bladder cancer. Oncotarget. 2015;6(12):10195–206. doi: 10.18632/oncotarget.3555 25991669 10.18632/oncotarget.3555 PMC4496349

[14] Liang H, Liu M, Yan X, Zhou Y, Wang W, Wang X, et al MiR-193a-3p functions as a tumor suppressor in lung cancer by down-regulating ERBB4. The Journal of biological chemistry. 2015;290(2):926–40. doi: 10.1074/jbc.M114.621409 25391651 10.1074/jbc.M114.621409 PMC4294520

[15] Williams. M, Kirschner. MB, Cheng. YY, Hanh. J, Weiss. J, Mugridge. N, et al MiR-193a-3p is a potential tumor suppressor in malignant pleural mesothelioma. Oncotarget. 2015;6(27):23480–95. doi: 10.18632/oncotarget.4346 26125439 10.18632/oncotarget.4346 PMC4695131

[16] Yu T, Li J, Yan M, Liu L, Lin H, Zhao F, et al MicroRNA-193a-3p and -5p suppress the metastasis of human non-small-cell lung cancer by downregulating the ERBB4/PIK3R3/mTOR/S6K2 signaling pathway. Oncogene. 2015;34(4):413–23. doi: 10.1038/onc.2013.574 24469061 10.1038/onc.2013.574

[17] Lv L, Li Y, Deng H, Zhang C, Pu Y, Qian L, et al MiR-193a-3p promotes the multi-chemoresistance of bladder cancer by targeting the HOXC9 gene. Cancer letters. 2015;357(1):105–13. doi: 10.1016/j.canlet.2014.11.002 25444900 10.1016/j.canlet.2014.11.002

[18] Yang Y, Zhou L, Lu L, Wang L, Li X, Jiang P, et al A novel miR-193a-5p-YY1-APC regulatory axis in human endometrioid endometrial adenocarcinoma. Oncogene. 2013;32(29):3432–42. doi: 10.1038/onc.2012.360 22907428 10.1038/onc.2012.360

[19] Tsai KW, Leung CM, Lo YH, Chen TW, Chan WC, Yu SY, et al Arm Selection Preference of MicroRNA-193a Varies in Breast Cancer. Scientific reports. 2016;6:28176 doi: 10.1038/srep28176 27307030 10.1038/srep28176 PMC4910092

[20] Lindstedt I, Lindgren MA, Andersson E, Engstrom W. The WT1 Gene–Its Role in Tumourigenesis and Prospects for Immunotherapeutic Advances. In vivo (Athens, Greece). 2014;28(5):675–82. 25189877

[21] Toska E, Roberts SG. Mechanisms of transcriptional regulation by WT1 (Wilms' tumour 1). The Biochemical journal. 2014;461(1):15–32. doi: 10.1042/BJ20131587 24927120 10.1042/BJ20131587 PMC8887836

[22] Moelans CB, Verschuur-Maes AH, van Diest PJ. Frequent promoter hypermethylation of BRCA2, CDH13, MSH6, PAX5, PAX6 and WT1 in ductal carcinoma in situ and invasive breast cancer. The Journal of pathology. 2011;225(2):222–31. doi: 10.1002/path.2930 21710692 10.1002/path.2930

[23] Qi XW, Zhang F, Yang XH, Fan LJ, Zhang Y, Liang Y, et al High Wilms' tumor 1 mRNA expression correlates with basal-like and ERBB2 molecular subtypes and poor prognosis of breast cancer. Oncology reports. 2012;28(4):1231–6. doi: 10.3892/or.2012.1906 22797561 10.3892/or.2012.1906

[24] Esquela-Kerscher A, Slack FJ. Oncomirs—microRNAs with a role in cancer. Nature reviews Cancer. 2006;6(4):259–69. doi: 10.1038/nrc1840 16557279 10.1038/nrc1840

[25] Yong FL, Law CW, Wang CW. Potentiality of a triple microRNA classifier: miR-193a-3p, miR-23a and miR-338-5p for early detection of colorectal cancer. BMC cancer. 2013;13:280 doi: 10.1186/1471-2407-13-280 23758639 10.1186/1471-2407-13-280 PMC3691634

[26] Nakano H, Yamada Y, Miyazawa T, Yoshida T. Gain-of-function microRNA screens identify miR-193a regulating proliferation and apoptosis in epithelial ovarian cancer cells. International journal of oncology. 2013;42(6):1875–82. doi: 10.3892/ijo.2013.1896 23588298 10.3892/ijo.2013.1896 PMC3699598

[27] Kwon JE, Kim BY, Kwak SY, Bae IH, Han YH. Ionizing radiation-inducible microRNA miR-193a-3p induces apoptosis by directly targeting Mcl-1. Apoptosis: an international journal on programmed cell death. 2013;18(7):896–909. 23546867 10.1007/s10495-013-0841-7

[28] Ma K, He Y, Zhang H, Fei Q, Niu D, Wang D, et al DNA methylation-regulated miR-193a-3p dictates resistance of hepatocellular carcinoma to 5-fluorouracil via repression of SRSF2 expression. The Journal of biological chemistry. 2012;287(8):5639–49. doi: 10.1074/jbc.M111.291229 22117060 10.1074/jbc.M111.291229 PMC3285337

[29] Tahiri A, Leivonen SK, Luders T, Steinfeld I, Ragle Aure M, Geisler J, et al Deregulation of cancer-related miRNAs is a common event in both benign and malignant human breast tumors. Carcinogenesis. 2014;35(1):76–85. doi: 10.1093/carcin/bgt333 24104550 10.1093/carcin/bgt333

[30] Uhlmann S, Mannsperger H, Zhang JD, Horvat EA, Schmidt C, Kublbeck M, et al Global microRNA level regulation of EGFR-driven cell-cycle protein network in breast cancer. Molecular systems biology. 2012;8:570 doi: 10.1038/msb.2011.100 22333974 10.1038/msb.2011.100 PMC3293631

[31] Gao XN, Lin J, Li YH, Gao L, Wang XR, Wang W, et al MicroRNA-193a represses c-kit expression and functions as a methylation-silenced tumor suppressor in acute myeloid leukemia. Oncogene. 2011;30(31):3416–28. doi: 10.1038/onc.2011.62 21399664 10.1038/onc.2011.62

[32] Leivonen SK, Sahlberg KK, Makela R, Due EU, Kallioniemi O, Borresen-Dale AL, et al High-throughput screens identify microRNAs essential for HER2 positive breast cancer cell growth. Molecular oncology. 2014;8(1):93–104. doi: 10.1016/j.molonc.2013.10.001 24148764 10.1016/j.molonc.2013.10.001 PMC5528509

[33] Liu L, Li Y, Liu S, Duan Q, Chen L, Wu T, et al Downregulation of miR-193a-3p inhibits cell growth and migration in renal cell carcinoma by targeting PTEN. Tumour biology: the journal of the International Society for Oncodevelopmental Biology and Medicine. 2017;39(6):1010428317711951. 28639901 10.1177/1010428317711951

[34] Fisher JN, Terao M, Fratelli M, Kurosaki M, Paroni G, Zanetti A, et al MicroRNA networks regulated by all-trans retinoic acid and Lapatinib control the growth, survival and motility of breast cancer cells. Oncotarget. 2015;6(15):13176–200. doi: 10.18632/oncotarget.3759 25961594 10.18632/oncotarget.3759 PMC4537007

