# Supplementary material for: MicroRNA-193a inhibits breast cancer proliferation and metastasis by downregulating WT1
Source: PLoS One. 2017 Oct 10;12(10):e0185565. doi: 10.1371/journal.pone.0185565 (PMC5634539; doi:10.1371/journal.pone.0185565)
Supplement: S1 Table — (DOC) [file pone.0185565.s001.DOC]

| Primer | Sequence |
| --- | --- |
| WT1-left (L) | 5′-CAA TCA GGG TTA CAG CAC GG-3′ |
| WT1-right (R) | 5′-GCT TGA ATG AGT GGT TGG GG-3′ |
| GAPDH-L | 5′- CCG GGA AAC TGT GGC GTG ATG G-3′ |
| GAPDH-R | 5′- AGG TGG AGG AGT GGG TGT CGC TGT T-3′ |
| LVX-miR-193a-L | 5′-GGA ATT CCG AGC GTC GTG TAA CCC TTG-3′ |
| LVX-miR-193a-R | 5′-GAC TAG TCG AGC GCA CCT CAC CAC TC-3′ |
| pMIR-WT1CDS-L | 5′-CCC AAG CTT CCC ACT TAC AGA TGC ACA GC-3′ |
| pMIR-WT1CDS-R | 5′-AGC TTT GTT TAA ACT TTC TGA CAA CTT GGC CAC C-3′ |
| Sh-WT1 | 5′-GCA GTG ACA ATT TAT ACC AAA-3′ |

**S1 Table. qRT-PCR Primer Sequences.**
