# Supplementary material for: MicroRNA-193a inhibits breast cancer proliferation and metastasis by downregulating WT1
Source: PLoS One. 2017 Oct 10;12(10):e0185565. doi: 10.1371/journal.pone.0185565 (PMC5634539; doi:10.1371/journal.pone.0185565)
Supplement: S1 Text — The base sequences in red are the target sites complemented by miR-193a-5p. (DOC) [file pone.0185565.s002.DOC]

**S1 Text. CDS of the human *WT1* isoform [EX(-)KTS(-) NM_000378]. The base sequences in red are the target sites complemented by miR-193a-5p.**

1 agctggggta aggagttcaa ggcagcgccc acacccgggg gctctccgca acccgaccgc

61 ctgtccgctc ccccacttcc cgccctccct cccacctact cattcaccca cccacccacc

121 cagagccggg acggcagccc aggcgcccgg gccccgccgt ctcctcgccg cgatcctgga

181 cttcctcttg ctgcaggacc cggcttccac gtgtgtcccg gagccggcgt ctcagcacac

241 gctccgctcc gggcctgggt gcctacagca gccagagcag cagggagtcc gggacccggg

301 cggcatctgg gccaagttag gcgccgccga ggccagcgct gaacgtctcc agggccggag

361 gagccgcggg gcgtccgggt ctgagccgca gcaaatgggc tccgacgtgc gggacctgaa

421 cgcgctgctg cccgccgtcc cctccctggg tggcggcggc ggctgtgccc tgcctgtgag

481 cggcgcggcg cagtgggcgc cggtgctgga ctttgcgccc ccgggcgctt cggcttacgg

541 gtcgttgggc ggccccgcgc cgccaccggc tccgccgcca cccccgccgc cgccgcctca

601 ctccttcatc aaacaggagc cgagctgggg cggcgcggag ccgcacgagg agcagtgcct

661 gagcgccttc actgtccact tttccggcca gttcactggc acagccggag cctgtcgcta

721 cgggcccttc ggtcctcctc cgcccagcca ggcgtcatcc ggccaggcca ggatgtttcc

781 taacgcgccc tacctgccca gctgcctcga gagccagccc gctattcgca atcagggtta

841 cagcacggtc accttcgacg ggacgcccag ctacggtcac acgccctcgc accatgcggc

901 gcagttcccc aaccactcat tcaagcatga ggatcccatg ggccagcagg gctcgctggg

961 tgagcagcag tactcggtgc cgcccccggt ctatggctgc cacaccccca ccgacagctg

1021 caccggcagc caggctttgc tgctgaggac gccctacagc agtgacaatt tataccaaat

1081 gacatcccag cttgaatgca tgacctggaa tcagatgaac ttaggagcca ccttaaaggg

1141 ccacagcaca gggtacgaga gcgataacca cacaacgccc atcctctgcg gagcccaata

1201 cagaatacac acgcacggtg tcttcagagg cattcaggat gtgcgacgtg tgcctggagt

1261 agccccgact cttgtacggt cggcatctga gaccagtgag aaacgcccct tcatgtgtgc

1321 ttacccaggc tgcaataaga gatattttaa gctgtcccac ttacagatgc acagcaggaa

1381 gcacactggt gagaaaccat accagtgtga cttcaaggac tgtgaacgaa ggttttctcg

1441 ttcagaccag ctcaaaagac accaaaggag acatacaggt gtgaaaccat tccagtgtaa

1501 aacttgtcag cgaaagttct cccggtccga ccacctg**aag accca**cacca ggactcatac

1561 aggtgaaaag cccttcagct gtcggtggcc aagttgtcag aaaaagtttg cccggtcaga

1621 tgaattagtc cgccatcaca acatgcatca gagaaacatg accaaactcc agctggcgct

1681 ttgaggggtc tccctcgggg accgttcagt gtcccaggca gcacagtgtg tgaactgctt

1741 tcaagtctga ctctccactc ctcctcacta aaaaggaaac ttcagttgat cttcttcatc

1801 caacttccaa gacaagatac cggtgcttct ggaaactacc aggtgtgcct ggaagagttg

1861 gtctctgccc tgcctacttt tagttgactc acaggccctg gagaagcagc taacaatgtc

1921 tggttagtta aaagcccatt gccatttggt gtggattttc tactgtaaga agagccatag

1981 ctgatcatgt ccccctgacc cttcccttct ttttttatgc tcgttttcgc tggggatgga

2041 attattgtac cattttctat catggaatat ttataggcca gggcatgtgt atgtgtctgc

2101 taatgtaaac tttgtcatgg tttccattta ctaacagcaa cagcaagaaa taaatcagag

2161 agcaaggcat cgggggtgaa tcttgtctaa cattcccgag gtcagccagg ctgctaacct

2221 ggaaagcagg atgtagttct gccaggcaac ttttaaagct catgcatttc aagcagctga

2281 agaaaaaatc agaactaacc agtacctctg tatagaaatc taaaagaatt ttaccattca

2341 gttaattcaa tgtgaacact ggcacactgc tcttaagaaa ctatgaagat ctgagatttt

2401 tttgtgtatg tttttgactc ttttgagtgg taatcatatg tgtctttata gatgtacata

2461 cctccttgca caaatggagg ggaattcatt ttcatcactg ggagtgtcct tagtgtataa

2521 aaaccatgct ggtatatggc ttcaagttgt aaaaatgaaa gtgactttaa aagaaaatag

2581 gggatggtcc aggatctcca ctgataagac tgtttttaag taacttaagg acctttgggt

2641 ctacaagtat atgtgaaaaa aatgagactt actgggtgag gaaatccatt gtttaaagat

2701 ggtcgtgtgt gtgtgtgtgt gtgtgtgtgt gtgtgtgttg tgttgtgttt tgttttttaa

2761 gggagggaat ttattattta ccgttgcttg aaattactgt gtaaatatat gtctgataat

2821 gatttgctct ttgacaacta aaattaggac tgtataagta ctagatgcat cactgggtgt

2881 tgatcttaca agatattgat gataacactt aaaattgtaa cctgcatttt tcactttgct

2941 ctcaattaaa gtctattcaa aaggaaaaaa aaaaaaa//
